# Patterns of Care and Follow-up of Patients with an Ipsilateral Breast Tumor Event After Breast-Conserving Surgery: A Multicenter Retrospective Cohort Study in the Netherlands

**DOI:** 10.1245/s10434-026-20080-x

**Published:** 2026-07-17

**Authors:** Lisca F. Wurfbain, Laura M. Tiels, Maurice J. C. van der Sangen, Coco J. E. F. Walstra, Marissa C. van Maaren, Birgit E. P. J. Vriens, C. Willemien. Menke-van der Houven van Oordt, Johanne G. Bloemen, Adri C. Voogd, Grard A. P. Nieuwenhuijzen, Desirée H. J. G. van den Bongard, Robert-Jan Schipper

**Affiliations:** 1 Department of Radiation Oncology, Amsterdam UMC Location Vrije Universiteit Amsterdam, Amsterdam, The Netherlands; 2 Cancer Center Amsterdam, Cancer Treatment and Quality of Life/Cancer Biology and Immunology, Amsterdam, The Netherlands; 3 Department of Surgery, Catharina Hospital Eindhoven, Eindhoven, The Netherlands; 4 Department of Radiotherapy, Catharina Hospital Eindhoven, Eindhoven, The Netherlands; 5 Department of Health Technology and Services Research, Technical Medical Centre, University of Twente, Enschede, The Netherlands; 6 Department of Research and Development, Netherlands Comprehensive Cancer Organisation (IKNL), Utrecht, The Netherlands; 7 Department of Internal Medicine, Catharina Hospital Eindhoven, Eindhoven, The Netherlands; 8 Department of Medical Oncology, Amsterdam UMC Location Vrije Universiteit Amsterdam, Amsterdam, The Netherlands; 9 Department of Epidemiology, Maastricht University, Maastricht, The Netherlands

**Keywords:** Breast cancer, Ipsilateral breast tumor event, Recurrence, Mastectomy, Breast-conserving therapy, Multidisciplinary treatment

## Abstract

**Purpose:**

This study investigated patterns of care and oncological outcomes in patients with ipsilateral breast tumor events (IBTE) after prior breast-conserving surgery (BCS) treated with curative intent.

**Methods:**

A retrospective cohort study was conducted on IBTE patients treated in 2016–2017 at 34 Dutch hospitals. Patient and characteristics, along with treatment details were collected and descriptive statistics were used for analysis. Overall survival (OS), disease-free survival (DFS) and breast cancer-specific survival (BCSS) were evaluated by using Kaplan-Meier analyses.

**Results:**

Of the 486 IBTE patients, 75.1% were diagnosed more than 5 years after the primary tumor, 87.4% had invasive breast cancer, and 12.6% DCIS. Distinguishing true recurrences from second primaries was not possible. Among invasive IBTE, 70 (14.6%) had primary DCIS and 350 (72.8%) an invasive primary tumor. Most IBTEs (54.9%) were T1, grade II (46.5%), and ER/PR+/HER2− (61.7%). Seventy-two (14.8%) patients received neoadjuvant systemic therapy. Salvage mastectomy was performed in 435 (89.5%) patients, while 51 (10.5%) underwent repeat BCS; 60.8% of these received postoperative whole-breast re-irradiation. Among mastectomy patients, 411 (94.5%) had prior radiotherapy; 13.1% received repeat radiotherapy, either alone (24.1%) or with hyperthermia (75.9%). Adjuvant systemic therapy was administered to 64.9%. After a median follow-up of 37 months, 83 (17.4%) developed a subsequent event: 33 (39.8%) second IBTE, 9 regional recurrences, and 41 (49.4%) distant metastases. Three- and five-year OS was 89% and 72.5%, respectively. Three- and five-year DFS was 85.5% and 69.9%, respectively. Three- and five-year BCSS was 95.1% and 86%, respectively.

**Conclusions:**

Salvage mastectomy was the predominant treatment for IBTE, although repeat BCS with re-irradiation is increasingly performed in selected low-risk patients. Survival outcomes were favorable. The study highlights the need for tumor re-assessment and emphasizes the variability in IBTE management, underscoring the need for standardized treatment guidelines.

Nowadays, the majority of patients with early-stage breast cancer is treated with breast-conserving therapy (BCT), consisting of breast-conserving surgery (BCS) and postoperative whole breast irradiation (WBI), or partial breast irradiation (PBI) for low-risk patients. The risk of locoregional recurrence (LRR) after BCT is low, varying between 0.5–1% per year, totaling <5% over 5 years.^1–3^ However, owing to the aging population and improving breast cancer survival rates, the absolute number of patients at risk for an ipsilateral breast tumor event (IBTE) is increasing.^1–3^

The management of IBTE is multidisciplinary, due to its heterogeneous character, and is determined by various prognostic factors and previously received treatments.^1,4^ Also, breast cancer professionals have been shown to have individual preferences regarding treatment of IBTE.^5^ Furthermore, patient preferences play a role in shared treatment decisions.

The optimal extent of locoregional and systemic treatment in patients with an IBTE is unclear and depends on the risk assessment of the tumor.^1^ The standard treatment for an IBTE after previous BCT is salvage mastectomy,^1^ despite its substantial impact on psychological wellbeing and quality of life.^6^ Data have shown that a second BCS combined with partial breast re-irradiation could be an alternative to salvage mastectomy in low-risk (size <2 cm, ER+, HER2−, nonlobular, unicentric, clinically node-negative, interval ≥2 years) IBTE patients.^4,7–10^ In high-risk cases where postoperative re-irradiation is indicated, hyperthermia (which enhances the cytotoxic effects of radiotherapy by heating tumor tissue) represents an additional treatment modality available in specialized centers.^11,12^ Stratifying patients by risk appears to be of meaningful value in guiding treatment decisions. While physicians have expressed willingness to perform second BCT,^5,13^ the actual implementation of this alternative approach in clinical practice and the variation in treatment patterns across different settings remains to be determined.

Beyond risk stratification, the biological nature of the IBTE itself is an important consideration. Discordance in tumor grade and receptor status between the primary tumor and IBTE may occur secondary to clonal evolution and tumor heterogeneity.^14–16^ DNA profiling can differentiate between a true recurrence (TR) or to a second primary tumor (SP), although it is indicated only when results directly impact treatment decisions.^15,17^ In clinical practice, the decision is commonly based on histological features, tumor location (e.g., quadrant), and the time interval between the primary tumor and the IBTE.^1^ Understanding these discordances and their potential impact on treatment decisions and prognosis is important for optimizing patient care; retrospective studies suggest that certain tumor characteristics may influence outcomes.^18^

Current guidelines report the heterogeneity and provide recommendations for management of IBTE patients; yet emerging evidence suggests that less intensive treatment approaches may be appropriate for certain subgroups. However, it remains unclear how widely these less intensive approaches are adopted in clinical practice, and significant knowledge gaps persist regarding which patients are most suitable for alternative treatment strategies. Large patient cohorts are necessary to evaluate current treatment patterns and identify prognostic factors that can inform evidence-based clinical decision-making in this diverse patient population.

The purpose of this large, retrospective multicenter study was to evaluate current patterns of care of patients with an IBTE after previous BCS treated with curative intent, to evaluate the characteristics of both primary tumor and IBTE, and to assess 3- and 5-year disease-free survival (DFS), breast cancer-specific survival (BCSS), and overall survival (OS).

## Methods

### Patients

In this multicenter retrospective cohort study, patients diagnosed with an IBTE after previous BCT for ductal carcinoma in situ (DCIS) or invasive breast cancer in 2016 and 2017 were selected from the Dutch Nationwide Pathology Databank.^19^ Patients had to be treated with curative intent for their IBTE. The search yielded 4291 cases. After manual screening of the pathology reports and to eliminate nonapplicable cases or exclusion due to incorrect use of codes, 1494 patients were considered eligible. In total, 34 of 72 hospitals participated in the present study, resulting in a final cohort of 486 patients. Data on tumor characteristics, locoregional and systemic treatment, and short-term follow-up were collected from patient files by using electronic case report files (eCRFs) up to January 2022. Because of the retrospective nature of this study, the need for informed consent was waived by the medical ethical committee of Catharina Hospital Eindhoven (decision number W18.148).

### Definitions and Outcomes

An IBTE was defined as any type of recurrent tumor growth in a previously preserved breast after primary breast cancer or DCIS. No distinction was or could be made between ipsilateral TR or second SP tumors. An IBTE was considered as a low-risk tumor if the following criteria were fulfilled: size <2 cm, ER receptor-positive, HER2-negative, nonlobular type, unicentric, clinically node-negative with an interval between primary tumor diagnosis and IBTE of at least 2 years. These criteria were based on existing literature on repeat BCT for IBTE.^1,4,7^ All other IBTEs not meeting these low-risk criteria were classified as nonlow-risk tumors. The type of invasive or noninvasive breast cancer was documented, and the tumors were graded by using the modified Bloom and Richardson grading system. Estrogen and progesterone receptor statuses were evaluated through immunohistochemistry, with tumors showing ≥10% expression classified as positive according to the Dutch guidelines.^20^ HER2 status was determined by immunohistochemistry, with fluorescence (chromogenic) in situ hybridization (FISH) performed for cases with equivocal (2+) immunohistochemistry results.^21^ Definitions of the events were based on the Maastricht Delphi consensus.^14^ A local recurrence (LR) in oncological follow-up after IBTE treatment was defined as recurrent (epithelial) tumor growth or DCIS in the ipsilateral breast, or skin and subcutaneous tissue of the ipsilateral thoracic wall. A regional recurrence (RR) was defined as recurrent disease in regional lymph nodes, including ipsilateral intramammary nodes, axillary nodes, and infraclavicular or supraclavicular nodes. A distant recurrence (DR) was defined as recurrent disease in any location except the ipsilateral breast, contralateral breast, or ipsilateral regional lymph nodes. Disease in lymph nodes other than ipsilateral regional lymph nodes was considered as distant metastasis.^14,22^ Tumor growth in the contralateral breast was defined as a second primary tumor. During the follow-up after curative treatment of the IBTE, events were considered synchronous if they were diagnosed within 3 months from each other. Disease-free survival was defined as the time between date of IBTE surgery and date of first event diagnosis. Breast cancer-specific survival was defined as the time between IBTE surgery and date of breast-cancer specific death. Overall survival was defined as the time between date of IBTE surgery and date of death or last observation. All survival rates were cut off at 3 and 5 years.

### Statistics

Descriptive statistics were used to describe patient-, tumor-, and treatment-related characteristics for both the primary tumor and the IBTE, including median and range, or count and percentage, as appropriate. A Sankey diagram was designed for visual presentation of the concordance/discordance between receptor statuses of the primary invasive tumor and the invasive IBTE. (Neo)adjuvant systemic treatment was described based on receptor status separately. Additionally, descriptive statistics were used to analyze occurrence of subsequent events. Kaplan-Meier analysis were performed to analyze the 3- and 5-year DFS, BCSS, and OS with 95% confidence intervals (CIs) after curative IBTE treatment. A log-rank test was performed to compare the survival of the predefined low- and high risk group. All statistical analyses were conducted by using IBM SPSS Statistics version 28.0.

## Results

### Patients

**Fig. 1:**
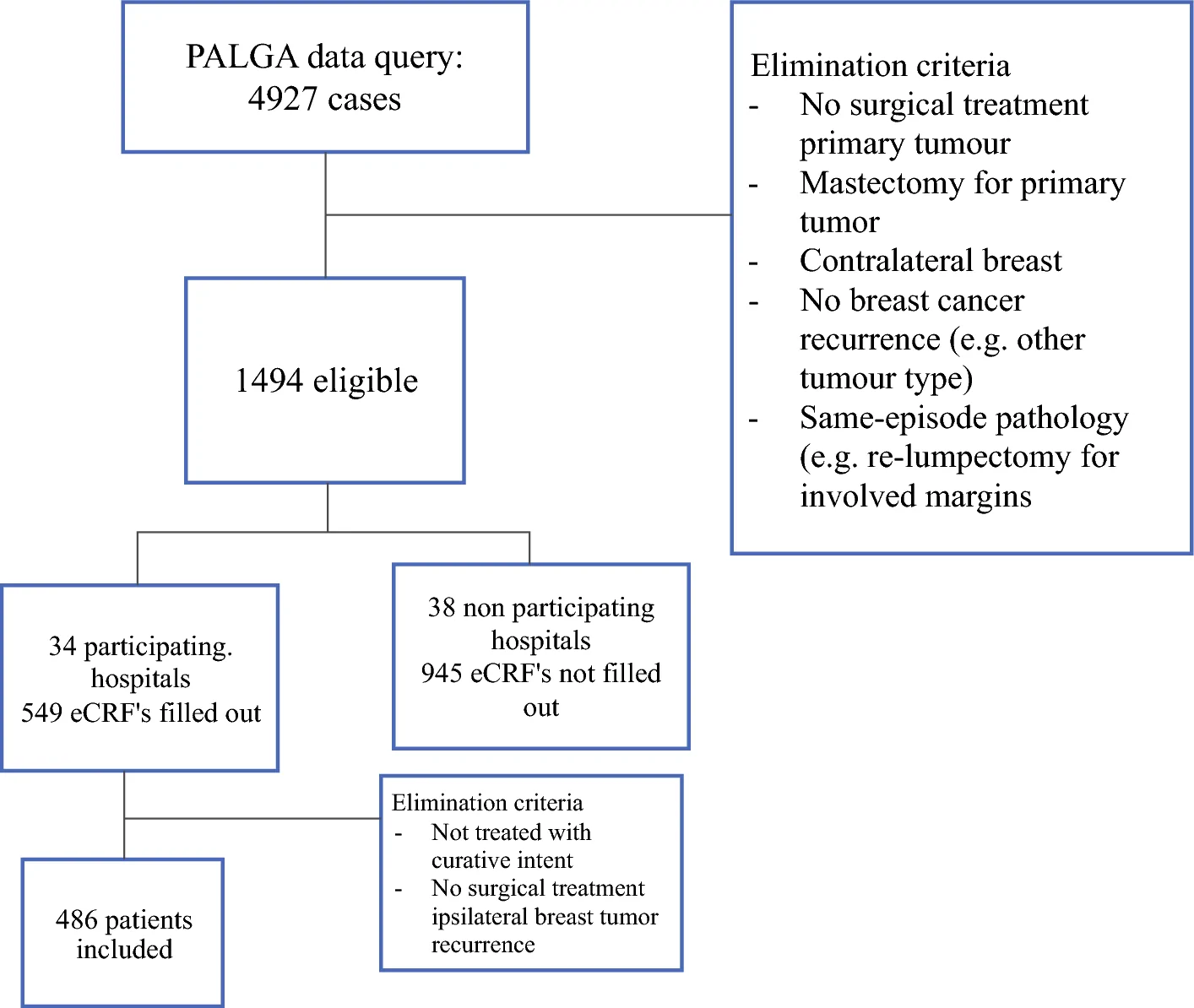
Flowchart inclusion

**Fig. 2:**
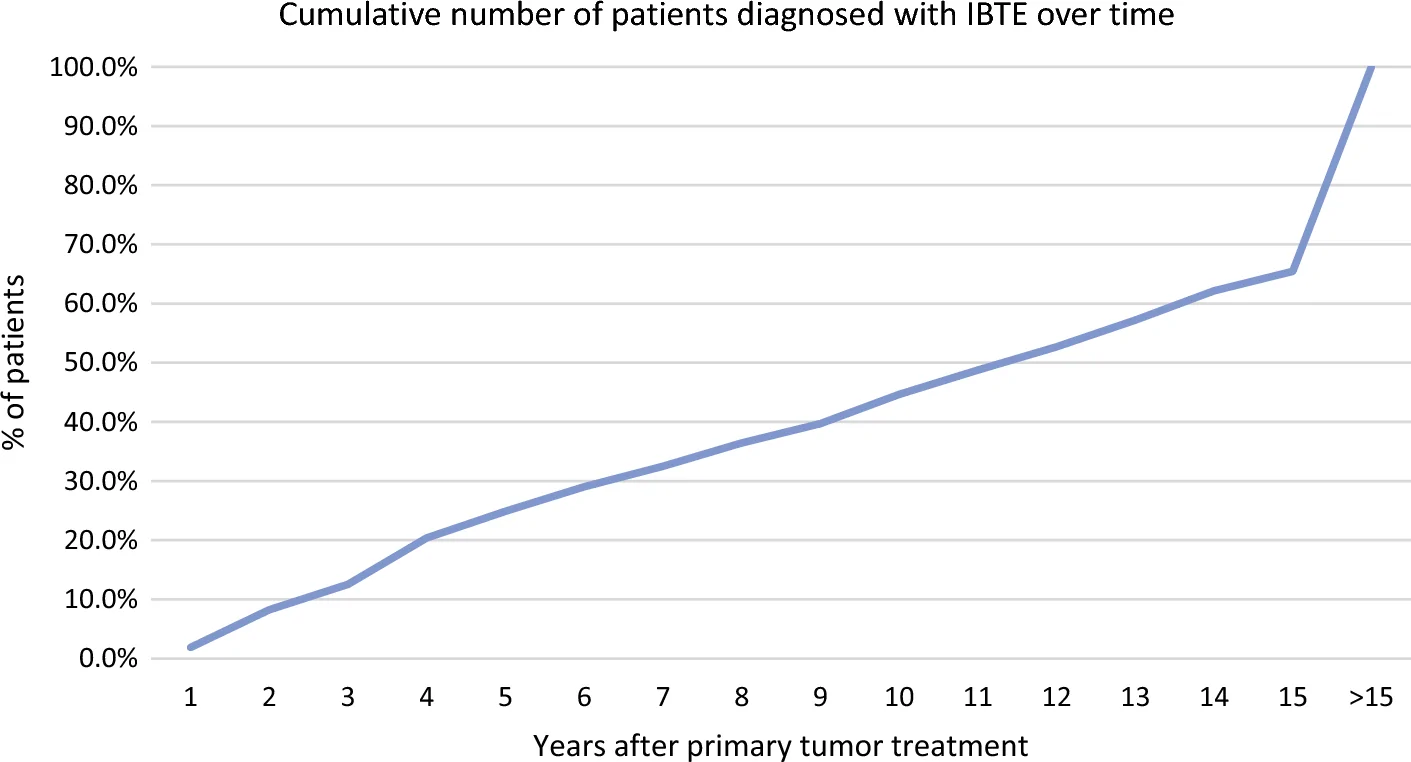
Time between the diagnosis of the primary breast cancer and IBTE

A total of 486 patients were included (Fig. 1). The median (range) age at diagnosis of the primary breast cancer and IBTE was 55 years (range 17–90 years), and 68 years (range 26–92 years), respectively. Treatment of the primary tumor took place between 1983 and 2016. The median time between diagnosis of the primary breast cancer and IBTE was 11 years (IQR 5–17 years). In total, 121 (24.9%) were diagnosed with IBTE within 5 years after the primary tumor diagnosis (Fig. 2). Among all included patients, 285 (58.6%) were diagnosed with IBTE during regular follow-up visits and 201 (41.4%) by the patients themselves having symptoms. Of the 124 (25.5%) IBTEs diagnosed within 5 years after treatment for the primary breast cancer, 93 (75%) were diagnosed at regular follow-up compared with 192 (53%) of the IBTEs diagnosed more than 5 years after treatment of the primary tumor (*p* < 0.01). In total, 198 patients (40.7%) were staged with PET-CT for their IBTE. More details on preoperative imaging strategies of this cohort is described elsewhere.^23^

### Tumor Characteristics of Primary Tumor and IBTE

Of 54 patients with ductal carcinoma in situ (DCIS) as IBTE, 28 had also a DCIS as primary tumor. Of the 432 patients with invasive IBTEs, 43 (9.9%) had a DCIS as primary tumor. Table 1 shows tumor characteristics for both the primary tumor and the IBTE. The majority of the primary tumors (56.6%) was clinically staged as T1, followed by T2 (19.5%). Most primary tumors were Bloom-Richardson grade II (30.2%), while in 32.5% the tumor grade was missing. ER/PR status was positive in 49% of the primary tumors. The HER2 status was positive in 5.8% of the patients, but unknown in 54.9%. The complete primary tumor receptor status (ER/PR/HER2) was unknown in 41.2% of the primary tumors.

**Table 1:** Patient, tumor, and treatment characteristics of the IBTE and the primary tumor

	Primary tumor (*n* = 486)	IBTE (*n* = 486)
Age, years	55 (17–90)	68 (26–92)
< 35	24 (4.9)	1 (0.2)
35–59	291 (59.9)	62 (12.8)
60–69	114 (23.5)	114 (23.5)
> 70	57 (11.7)	309 (63.6)
Time from primary surgery to IBTE diagnosis		
Median, months (range)	*n.a.*	133.5 (4–395)
< 36 months	*n.a.*	69 (14.2)
36–60 months	*n.a.*	55 (11.3)
> 60 months	*n.a.*	362 (74.5)
Detection of IBTE		
Follow-up screening	*n.a.*	285 (58.6)
Self-reported	*n.a.*	201 (41.4)
Axillary surgery		
No surgical axillary staging	52 (10.7)	77 (15.8)
pN0(sn)	223 (45.9)	326 (67.1)
pN1mi(sn), no axillary RT	1 (0.2)	8 (1.6)
pN1mi(sn), axillary RT	6 (1.2)	2 (0.4)
pN1(sn), axillary RT	8 (1.6)	6 (1.2)
pN1(sn), no axillary RT	1 (0.2)	14 (2.9)
pN1(sn), cALND	34 (7.0)	5 (1.0)
ALND	157 (32.3)	47 (9.7)
Unknown	4 (0.8)	1 (0.2)
Tumor size		
Tis	71 (14.6)	54 (11.1)
T1	275 (56.6)	267 (54.9)
T2	95 (19.5)	92 (18.9)
T3	2 (0.4)	18 (3.6)
T4	3 (0.6)	24 (4.9)
Unknown	40 (8.2)	31 (6.4)
Tumor grade		
I	94 (19.3)	79 (16.3)
II	147 (30.2)	226 (46.5)
III	87 (17.9)	140 (28.8)
Unknown	158 (32.5)	41 (8.4)
Histologic subtype		
Ductal (NST)	291 (59.9)	323 (66.5)
Lobular	38 (7.8)	53 (10.9)
Other	25 (5.1)	51 (10.5)
Unknown	61 (12.6)	5 (1.0)
DCIS	71 (14.6)	54 (12.6)
Receptor status		
ER/PR+HER2−	158 (32.5)	300 (61.7)
ER/PR+HER2+	14 (2.9)	32 (6.6)
ER/PR−HER2+	11 (2.3)	12 (2.5)
ER/PR−HER2−	32 (6.6)	63 (10.0)
Not applicable (i.e. DCIS)	71 (14.6)	54 (11.1)
Unknown	200 (41.2)	25 (5.1)
Surgical treatment		
Lumpectomy	486 (100.0)	51 (10.5)
Mastectomy	*n.a.*	435 (89.5)
Neoadjuvant treatment		
None	462 (95.1)	414 (85.2)
Chemo-(± target) therapy	12 (2.5)	49 (10.1)
Endocrine therapy	5 (1.0)	27 (5.6)
Radiotherapy	7 (1.4)	*n.a.*
Adjuvant treatment		
None	320 (65.8)	211 (43.3)
Chemo-(± target) therapy	86 (17.7)	89 (18.3)
Endocrine therapy	86 (17.7)	229 (47.1)
Radiotherapy breast		
Yes	442 (90.9)	76 (15.6)
No	44 (9.1)	410 (84.4)

Data presented as median and range or count and percentage

*IBTE* ipsilateral breast tumor recurrence; *n.a.* not applicable; *SN* sentinel node; *RT* radiotherapy; *ALND* axillary lymph node dissection; *cALND* complete axillary lymph node dissection; *NST* no specific type; *ER* estrogen receptor; *PR* progesterone receptor; *HER2* human epidermal growth factor 2; *DCIS* ductal carcinoma in situ

The majority of the IBTEs (*n* = 267; 54.9%) were clinically staged as T1, followed by T2 (*n* = 92; 18.9%). IBTEs were predominantly grade II 226 (46.5%) and grade III 140 (28.8%). The majority of the invasive IBTEs 300 (61.7%) were ER/PR+/HER2−. Of all patients with invasive IBTE, 133 (27.4%) were classified as low-risk (see Methods section). ER/PR and HER2 status were both unknown in 25 (5.1%) of the patients with an invasive IBTE.

### Differences in Receptor Statuses Between Primary Tumor and IBTE

**Fig. 3:**
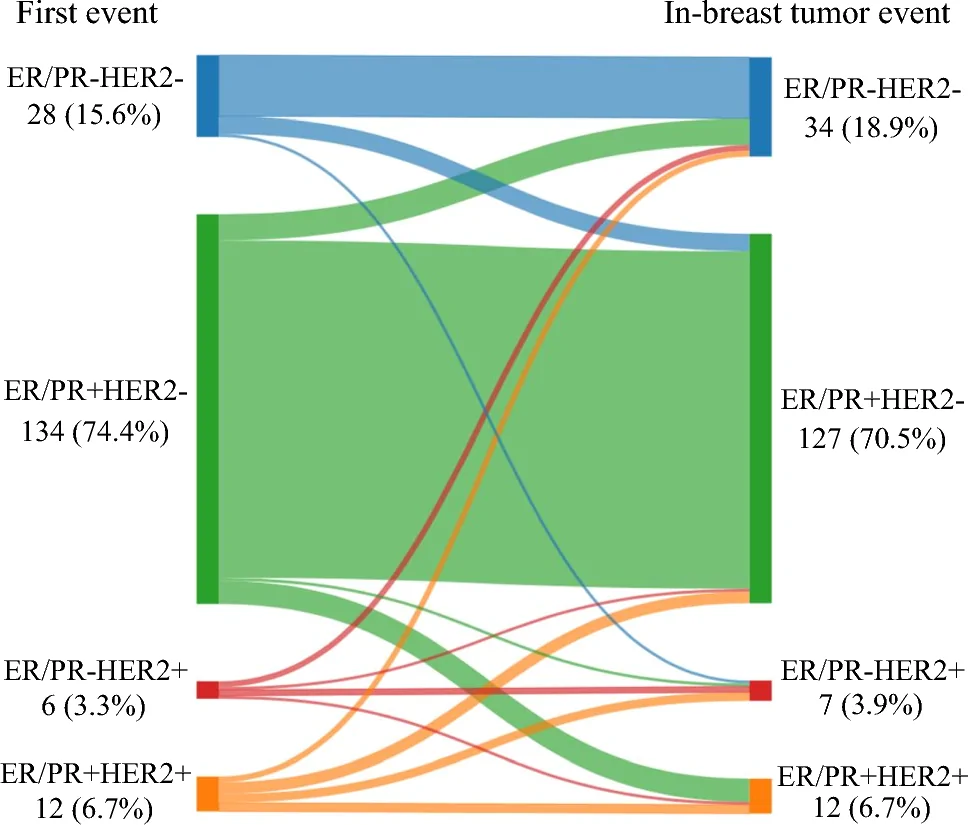
Differences between receptor statuses for the primary tumor and IBTE. *PRIM* primary tumor; *IBTE* ipsilateral breast tumor recurrence (*n* = 180)

Figure 3 shows the differences in receptor statuses between the primary invasive tumor and the IBTE invasive tumor (*n* = 180). In 21.1% of the patients, the receptor status was discordant. In the 273 patients with an invasive tumor of whom the ER/PR status of the primary invasive tumor and IBTE was available, a discordant result was found in 17.2%.

### Treatment of IBTE

#### Locoregional treatment

Among 486 included patients with IBTE, 51 (10.5%) were treated with repeat BCS and 435 (89.5%) with salvage mastectomy. Of the 435 patients, 19.3% received an immediate breast reconstruction after salvage mastectomy. In 74.5% of the patients with IBTE, axillary surgery consisted of sentinel lymph node biopsy (SLNB), of which 90.1% were tumor-negative. A complete axillary lymph node dissection (ALND) was performed in 1% of the patients with an IBTE, following a positive sentinel node procedure. Primary ALND was performed in 9.5% of the patients with IBTE.

Of the 51 patients treated with repeat BCS, 31 (60.8%) received postoperative radiotherapy for their primary tumor. Of these 31 patients, six (19.4%) were treated with PBI after surgery for their IBTE. Of the remaining 20 patients not treated with radiotherapy for the primary tumor, 60% received WBI for their IBTE. More details are presented in Table 2.

**Table 2:** Radiotherapy treatment after salvage mastectomy for IBTE compared with primary radiotherapy treatment

Salvage mastectomy (*n* = 435)	IBTE no RTX	IBTE thoracic wall	IBTE RTX + hyperthermia	Total
Primary no RTX	20 (4.6)	4 (0.9)	0 (0)	24 (5.5)
Primary WBI	357 (82.1)	13 (3.0)	41 (9.4)	411 (94.5)
Total	377 (86.7)	17 (3.9)	41 (9.4)	435 (100)

Data presented as count and percentage

*IBTE* ipsilateral breast tumor event

In IBTE patients treated with salvage mastectomy, 411 (94.5%) received radiotherapy for their primary tumor of whom 54 (13.1%) received re-irradiation for IBTE, either alone (24.1%) or in combination with hyperthermia (75.9%). Of 24 (5.5%) patients not treated with WBI for the primary tumor, four (16.7%) received radiotherapy for IBTE. More details are presented in Table 3.

**Table 3:** Radiotherapy treatment after repeat BCS for IBTE compared with primary radiotherapy treatment

Repeat BCS (*n* = 51)	IBTE no RTX	IBTE breast	IBTE RTX + hyperthermia	Total
Primary no RTX	8 (15.7)	12 (23.5)	0 (0)	20 (39.2)
Primary WBI	25 (49)	5 (9.8)	1 (2)	31 (60.8)
Total	33 (64.7)	17 (33.3)	1 (2)	51 (100)

*IBTE* ipsilateral breast tumor event; *BCS* breast-conserving surgery

### (Neo-)adjuvant systemic therapy

Of all 486 patients with IBTE, 76 (15.7%) received neoadjuvant systemic therapy, of whom, 49 (64.5%) received neoadjuvant chemotherapy +/− targeted therapy and 27 (35.5%) endocrine therapy. Furthermore, 65.4% of all patients with IBTE were treated with adjuvant systemic therapy. More specifically, 57.2% of the patients with a triple negative IBTE and 19% of the ER/PR+HER2− IBTEs were treated with chemotherapy, whereas 58.3% of the patients with ER/PR−HER2+ and 62.5% of the patients with ER/PR+HER2+ IBTEs were treated with chemotherapy +/− targeted therapy. Furthermore, 66.6% of the ER/PR+ IBTEs were treated with endocrine therapy.

### Three- and five-year survival rates

**Fig. 4:**
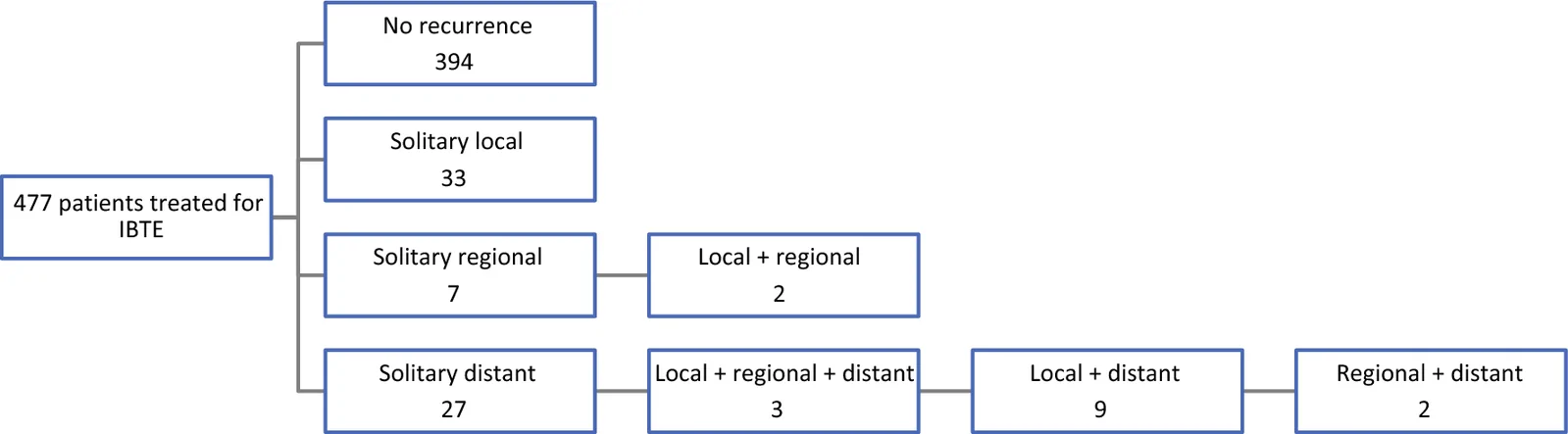
Details on type of recurrence after treatment of IBTE

**Fig. 5:**
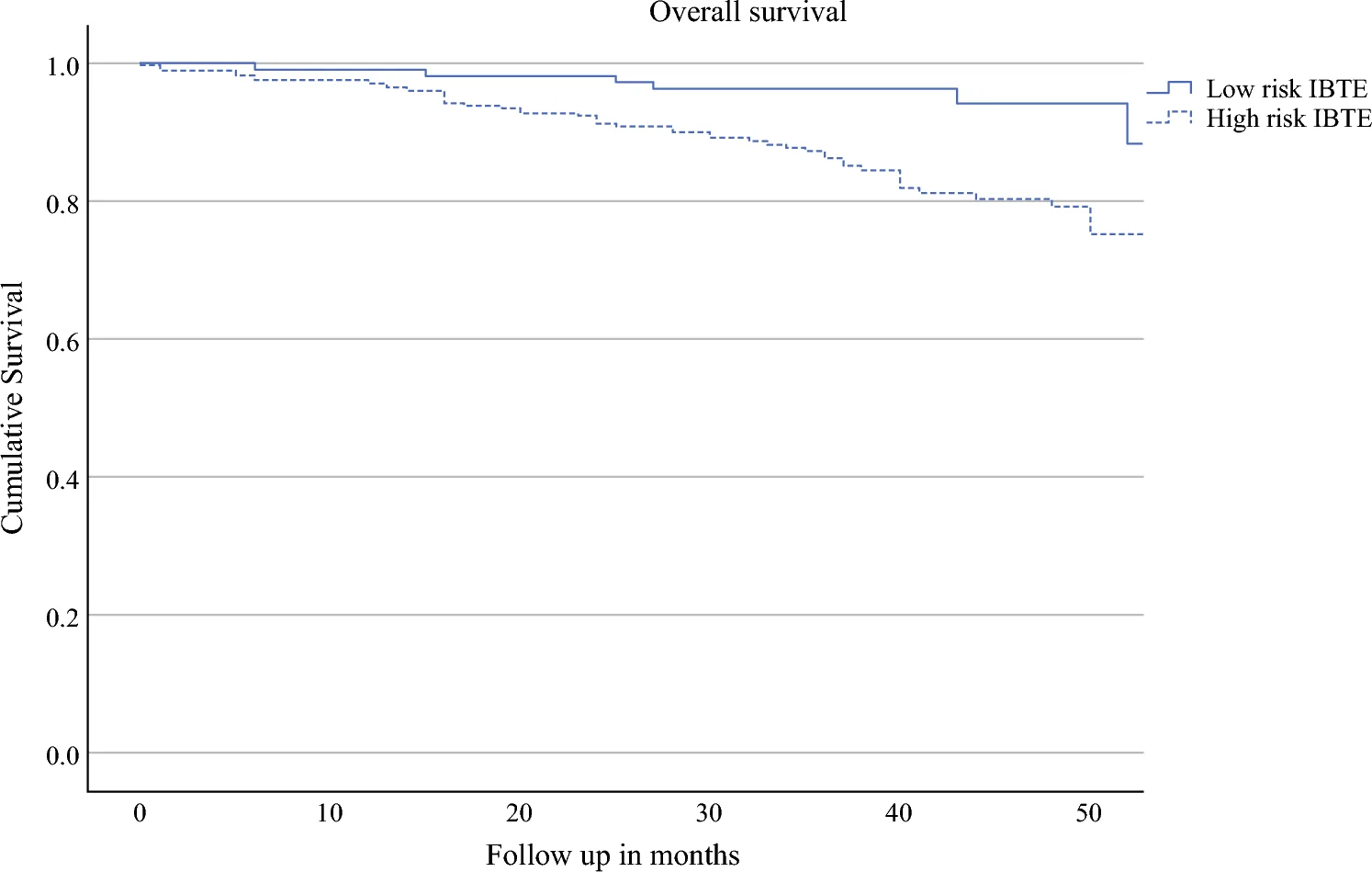
Survival of patients treated for IBTE

A total of 477 patients were included in these analyses, after excluding nine (1.9%) patients without data on follow-up. The median follow-up of patients after IBTE treatment was 37 months (range 1–72). In total, 83 (17.4%) of the patients with IBTE were diagnosed with a subsequent breast cancer-related event (Fig. 4). Of these, 33 (39.8%) had a solitary, second local recurrence, while 41 (49.4%) developed distant metastases. During follow-up 67 patients (14%) died, of whom 29 (43.3%) due to metastatic disease and 21 (31.3%) from nonbreast cancer-related causes. For 17 (25.4%) patients, the cause of death was unknown. The 3-year DFS was 85.5% (95% CI 83.3–86.7), and the 5-year DFS was 69.9% (95% CI 64.7–75.1). The 3-year and 5-year BCSS were 95.1% (95% CI 94.1–96.1) and 86% (95% CI 79.9–92.1), respectively. Estimated 3- and 5-year OS rates were 89% (95% CI 86.1–91.9) and 72.5% (95% CI 60.9–84.1), respectively. More detailed information of survival according to IBTE treatment modality was reported in Table 4. Patients with low-risk IBTE showed a significantly better overall survival than patients with high-risk IBTE; 3- and 5-year survival rates were 96.3% and 75.7%, respectively, for the low-risk group and 86.2% and 69.4%, respectively, for the high-risk group (*p* = 0.006; Fig. 5).

**Table 4:** Three-year survival rates according to IBTE treatment modality

IBTE locoregional treatment	*N* (%)*	DFS (%, 95%CI)	BCSS (%, 95%CI)	OS (%, 95% CI)
BCS	33 (6.9%)	73.5% (65.4–81.6)	100%	84.2% (76.9–91.5)
BCT (BCS and (re)irradiation ± hyperthermia)	16 (3.4%)	92.9% (86–99.8)	92.9% (86–99.8)	85.7% (76.3–95.1)
SM	371 (77.8%)	88.2% (86.4–90)	96% (94.9–97.1)	90.9% (89.3–92.5)
SM and (re)irradiation ± hyperthermia	57 (11.9%)	68.6% (62.2–75)	87.1% (82.1–92.1)	78.5% (72.7–84.3)

*IBTE* ipsilateral breast tumor event; *DFS* disease-free survival; *BCSS* breast cancer-specific survival; *OS* overall survival; *BCS* breast-conserving surgery; *BCT* breast-conserving therapy; *SM* salvage mastectomy

^*^Based on *n* = 477

## Discussion

This retrospective cohort study was designed to provide a comprehensive overview of patient-, tumor-, and treatment-related characteristics and follow-up of IBTE patients in the Netherlands treated with a curative intent in 2016–2017. All patients were at least previously treated with BCS.

We found that 89.5% of patients with IBTE underwent salvage mastectomy after previous BCT, aligning with the international guidelines that recommend salvage mastectomy as the standard care for IBTE.^1,20^ Recent literature and ongoing trials provide increasing evidence that repeat BCT is a safe and feasible option for patients with low-risk IBTE, defined as up to 2 cm in diameter, ER+, N0.^4,7–10,24,25^ However, treating physicians report persistent concerns regarding oncological outcomes and toxicity.^26^ Furthermore, Seto et al. found that among patients who underwent repeat BCS for IBTE, many declined postoperative breast re-irradiation, potentially due to concerns about radiation-related toxicity and side effects.^19^ Conversely, physicians may be hesitant to recommend repeat BCS owing to concerns about treatment tolerability and potential complications from re-irradiation. This divergence between patient reluctance toward re-irradiation and physician caution regarding repeat BCS creates discrepancies in treatment preferences for IBTE management, with variations in practice patterns influenced by international differences and individual patient and professional preferences. The high percentage of salvage mastectomies in our cohort was in line with the treatment period of the patients, stating salvage mastectomy as standard care in the Dutch guidelines.^20^ More recent data should be investigated if patients are more frequently treated with a second BCT in case of a low-risk IBTE. However, recent treatment trends would have inadequate follow-up duration for reliable oncological outcome assessment. Prospective data are needed on the oncological safety of second BCT in patients with IBTE following previous BCT, because the majority of existing data have been obtained in single-arm, retrospective, monocenter studies.^4,5,7^

In patients with a clinically node-negative IBTE, a repeat sentinel lymph node biopsy (rSLNB) is feasible, but its additional value is debated.^27–30^ In clinically node-positive recurrent breast cancer, a combination of (neo)adjuvant systemic treatment followed by surgery and adjuvant radiotherapy is indicated.^1,31^

In the current study, after a 3-year follow-up period, only 17% of the patients presented with a new event; however, approximately one-third of recurrences was a subsequent IBTE. Notably, of the patients with IBTE undergoing mastectomy a minority received postmastectomy radiotherapy. While the literature supports the use of postmastectomy radiotherapy for high-risk tumors and underscores its importance in the treatment protocol, it is important to note that indications for postoperative re-irradiation in high-risk IBTE have been extrapolated from indications for postoperative radiotherapy in primary breast cancer treated with mastectomy. Despite this established rationale, our cohort underwent an unexpectedly low rate of radiotherapy.^1,31^ Potentially this discrepancy led to the frequent new IBTE event. Clearly, there is a need for clearer indications and more consistent application of radiotherapy in such cases. Differences in clinical practice regarding re-irradiation include variations in the incorporation of adjunctive therapies, such as hyperthermia, which may influence treatment outcomes and patient management strategies.^11,12^ A current multicenter, prospective cohort study conducted in nine Dutch hospitals, the RT-HYPE trial (clinicaltrials.gov NCT06452485), aims to collect real-world data on oncological outcomes, patient-reported toxicity, and quality of life in high-risk LRR breast cancer patients treated with postoperative re-irradiation with or without hyperthermia, or surgery only. This data will help to guide the shared decision-making between patients and professionals. Importantly, within the RT-HYPE project, the delivery of hyperthermia has been harmonized across participating centers to ensure treatment consistency; a dedicated manuscript describing this harmonization is currently in preparation and was presented at the ESTRO 2026 annual congress.

In our cohort, 41 of the 477 patients with IBTE experienced a distant event during follow-up. This substantial risk of distant progression of disease highlights the importance of discussing the role of systemic treatment options for patients with IBTE. Current indications for (neo)adjuvant systemic treatment in IBTE remain unclear; few prospective clinical trials offer high-level evidence.^1,33^ The CALOR trial, which explored the role of chemotherapy and endocrine therapy following surgical excision of isolated locoregional recurrences (55% of which were IBTE), highlighted variability in outcomes related to ER status.^32^ Patients with hormone receptor-positive breast cancers, which represent a substantial proportion of IBTE cases, had clear survival benefit of adjuvant endocrine therapy; however, the benefit of chemotherapy was predominantly found in hormone receptor-negative tumors. The current treatment approaches extrapolate from the guidelines for primary breast cancer treatment and consider the molecular subtype—the extent of the IBTE and the patient’s previous treatment history—to decide whether the patient is a candidate for chemotherapy, HER2-targeted therapy, and/or endocrine therapy.^34,35^ However, the lack of evidence including regarding the optimal duration and sequencing of hormone therapy underscores the necessity for further research.

The observed low prevalence of HER2-positive tumors (5.8%) in our cohort compared with the expected 10–15% in primary breast cancer may reflect the higher propensity of HER2-positive tumors for distant metastasis, potentially precluding local recurrence.^36,37^ While HER2-positive tumors historically carried increased local recurrence risk, the introduction of trastuzumab in the Netherlands in 2006 likely altered this pattern. The high proportion of unknown HER2 status (54.9%) predominantly reflects the pre-trastuzumab era, when HER2 testing was not routinely performed and targeted therapy was unavailable.

Receptor and grade discordance between primary and recurrent invasive tumors occurs in a substantial number of patients and poses significant challenges for treatment decision-making.^6,7,30,31^ This discordance may arise from several mechanisms, including clonal evolution in TRs or the development of biologically distinct SPs. While DNA profiling can theoretically help distinguish between these scenarios, it is primarily indicated only when it directly impacts treatment decisions.^7,8^ Similar patterns of receptor conversion are well-documented in the metastatic setting, highlighting the biological complexity of tumor evolution.^38,39^ Given the treatment implications of receptor discordance, comprehensive receptor status assessment should be performed for invasive IBTEs when treatment decisions may be influenced by the results. This approach ensures that systemic therapy recommendations, particularly regarding hormonal and targeted treatments, are based on current tumor characteristics rather than assumptions derived from the primary tumor profile, as emphasized by recent studies including Meegdes et al.^30^

In our study, the median time between primary breast cancer diagnosis and IBTE was 11 years; 74.5% of IBTE cases occurred more than 5 years after the primary diagnosis. This contrasts with existing literature, where the majority of IBTE cases tend to occur within the first 2 years postdiagnosis.^15,36,40^ The longer median interval observed in our cohort reflects a more selective patient population, potentially influenced by factors, such as treatment protocols or patient follow-up. Additionally, this selective nature may be attributed to the fact that patients were derived from only 34 hospitals. Literature demonstrates that ER+/PR+/HER2− tumors typically recur later compared to triple negative tumors.^15^ This pattern is consistent with our cohort findings, where the majority of invasive IBTEs demonstrated ER/PR+/HER2− status. Interestingly, in our study 58.6% of all IBTE cases were detected during regular follow-up screening and 41.4% were detected by the patients themselves. However, for IBTE diagnosed within the 5-year follow-up period, 75% were detected during regular screening and only 25% by the patients, indicating that regular follow-up in the first years after treatment may be effective in detecting IBTE early.

The strength of this study lies in its description in the large cohort of a heterogeneous group of patients as seen in daily practice and in its exploration of treatment variation, offering valuable insights into current practice patterns. Furthermore, we gathered extensive and detailed data on both tumor characteristics and treatment regimens, allowing for a more comprehensive analysis of factors that may influence outcomes. These strengths contribute to a deeper understanding of the heterogeneity in IBTE management.

Our study has several limitations. First, this was an exploratory retrospective study, and the research population was a selected cohort from multiple hospitals in the Netherlands, which may limit the generalizability of the findings. A relatively large proportion of data were missing regarding the primary breast cancer characteristics, such as receptor status and tumor grade. Many primary tumors were diagnosed several decades ago, at times when the registration was not as complete as it is now. Furthermore, diagnostic methods and treatment strategies have evolved over the study period, meaning that not all patients in this cohort have benefited from the most recent treatment options. The relatively short follow-up period after diagnosis of IBTE, especially for luminal A-type breast cancer, further limits the assessment of long-term outcomes. Finally, owing to the considerable heterogeneity of the study population across tumor characteristics, treatment modalities, and participating centers, we were unable to perform a multivariable analysis of predictors of recurrence, which limits the ability to draw conclusions about individual prognostic factors in this cohort.

Further research with more extensive and prospective data on the outcomes of second BCT and the potential need for systemic therapy in IBTE is needed to validate our findings and support more informed clinical decision-making. Future perspectives for IBTE management should focus on developing more sophisticated risk stratification tools and personalized treatment approaches. Improved methods for distinguishing between TR and SP tumors through advanced genomic profiling may enhance treatment decision-making when clinically relevant.^6,7,30,31^ Risk stratification tools may further support individualized treatment selection, and several prognostic scores have been proposed to guide shared decision-making in IBTE management. One such tool is the TAM score, which was developed to predict outcomes following repeat BCT and incorporates tumor size, axillary nodal status, and molecular subtype to stratify patients by recurrence risk.^7^ To our knowledge, the TAM score currently represents the only available instrument that provides some guidance on treatment direction and outcome expectations in this setting. While we do not validate the TAM score in the present study and prospective validation in larger cohorts is still needed, we consider it a potentially valuable reference point for clinical decision-making in identifying patients suitable for second BCT versus salvage mastectomy. Building upon the TAM score framework, we recommend the importance of classifying patients into the following groups for IBTE management: 1) low-risk tumors, where either salvage mastectomy or second breast-conserving therapy may be offered if technically feasible and desired by the patient; 2) high-risk tumors, requiring salvage mastectomy with postoperative re-irradiation with/without hyperthermia; and 3) an intermediate-risk group, representing the largest proportion of patients, who would be candidates for salvage mastectomy alone. Furthermore, all groups may receive (neo)adjuvant systemic therapy based on individual tumor and patient characteristics. This stratified approach could optimize treatment selection while balancing oncological safety with quality of life considerations. A structured follow-up protocol is essential for monitoring subsequent events after a first IBTE, incorporating clinical examinations, imaging studies, and tumor markers.

## Conclusions

Salvage mastectomy remained the predominant surgical approach for IBTE patients treated with curative intent in the Netherlands during 2016–2017, although repeat BCS with re-irradiation was performed in selected patients. Our findings reveal the heterogeneity in IBTE management. The observed receptor discordance between primary tumors and IBTEs emphasizes the importance of re-determining tumor characteristics to guide appropriate (systemic) therapy decisions. The complexity and variation in current practice patterns underscore the critical need for better defining low-risk patient populations who may safely benefit from breast-conserving approaches. Future studies should focus on establishing the oncological safety of repeat BCT, developing standardized patient selection criteria, and creating evidence-based treatment protocols to optimize individualized care for IBTE patients.
